# How to resolve confusion in the clinical setting for the diagnosis of heterozygous *COL4A3* or *COL4A4* gene variants? Discussion and suggestions from nephrologists

**DOI:** 10.1007/s10157-020-01880-1

**Published:** 2020-03-30

**Authors:** Aya Imafuku, Kandai Nozu, Naoki Sawa, Koichi Nakanishi, Yoshifumi Ubara

**Affiliations:** 1 Nephrology Center, Toranomon Hospital, 2-2-2, Toranomon, Minato-ku, Tokyo, 105-8470 Japan; 2 Department of Pediatrics, Kobe University Graduate School of Medicine, 7-5-1 Kusunoki-cho, Chuo-ku, Kobe, 650-0017 Japan; 3 Okinaka Memorial Institute for Medical Research, Toranomon Hospital, 2-2-2, Toranomon, Minato-ku, Tokyo, 105-8470 Japan; 4 Department of Child Health and Welfare (Pediatrics), Graduate School of Medicine, University of the Ryukyus, 207 Uehara, Nishihara-cho, Okinawa 903-0215 Japan

**Keywords:** Thin basement membrane nephropathy, Autosomal dominant Alport syndrome, Heterozygous *COL4A3* or *COL4A4* variant

## Abstract

Both thin basement membrane nephropathy (TBMN) and autosomal dominant Alport syndrome (ADAS) are types of hereditary nephritis resulting from heterozygous mutations in *COL4A3* or *COL4A4* genes. Although TBMN is characterized by hematuria and thinning of the glomerular basement membrane (GBM) with excellent renal prognosis, some patients develop end-stage renal disease (ESRD) later in life. In contrast, although AS is characterized by progressive nephropathy with lamellation of the GBM, there are some patients diagnosed with ADAS from a family history of ESRD but who only suffer from hematuria with GBM thinning. These findings indicate a limitation in distinction between TBMN and ADAS. Diagnosis of AS is significant because it facilitates careful follow-up and early treatment, whereas diagnosis of TBMN can underestimate the risk of ESRD. However, some experts are against using the term ADAS as the phenotypes of heterozygous variants vary from no urinary abnormality to ESRD, even between family members with the same mutations, indicating that unknown secondary factors may play a large role in the disease severity. These diagnostic difficulties result in significant confusion in clinical settings. Moreover, recent studies revealed that the number of patients with chronic kidney disease caused by these gene mutations is far higher than previously thought. The aim of this article is to review differing opinions regarding the diagnosis of heterozygous *COL4A3* or *COL4A4* variants, and to highlight the importance for nephrologists to recognize this disease, and the importance of the need to reclassify this disease to minimize the current confusion.

## Introduction

Thin basement membrane nephropathy (TBMN) and Alport syndrome (AS) are common hereditary kidney diseases caused by structural abnormalities in the type IV collagen α-chains of glomerular basement membrane (GBM) [1, 2]. The concept of two diseases was originally proposed based on clinicopathological features. Most individuals with TBMN present with hematuria, with or without mild proteinuria, and normal renal function with a diffuse thinning of the GBM. However, there are some patients with TBMN that develop end-stage renal disease (ESRD) in their later lives [3, 4]. In contrast, AS is defined as progressive renal failure with irregular thickening and lamellation of the GBM, accompanied by hearing loss and ocular abnormalities [1]. However, some patients with AS show renal impairment with only GBM thinning without lamellation, and lack of extrarenal manifestations [5–7]. These findings indicated limitations in distinction between TBMN and AS based on clinicopathological features. After genetic analysis became available, it has been proven that both TBMN and autosomal dominant AS (ADAS) are caused by heterozygous mutations in either *COL4A3* or *COL4A4* genes [5, 8]. Although ADAS was thought to be an extremely rare disease and its disease concept was unclear for a long time, recent studies clarified genetic, clinical, and pathological features [6]. Furthermore, due to the rapid development of comprehensive genetic analysis technique using next-generation sequencing (NGS), it has been revealed that the number of patients with chronic kidney disease (CKD) caused by these gene mutations is far higher than previously expected [9–12], resulting in ongoing discussions regarding the diagnosis of this condition [13–17]. Here, we review the previous publications regarding TBMN and ADAS and discuss the diagnosis of cases with heterozygous *COL4A3* or *COL4A4* variants.

### Changes in the concept of TBMN

TBMN is a common hereditary nephritis which affects at least 1% of the population [2]. In the 1960s, several families with isolated hematuria were reported as benign familial hematuria (BFH) [18]. In 1973, thinning of the GBM by electron microscopy (EM) was observed in BFH families [19]. Whilst the authors stated that this condition could be labeled “benign” only after prolonged observation, the term “TBMN” has often been used synonymously with “BFH” until recently.

After the 1980s, some researchers reported that cases of TBMN were occasionally accompanied by severe proteinuria [20] or development of ESRD [21]. However, it was thought that proteinuria or renal dysfunction may be due to other coincidental factors such as glomerulonephritis or hypertension, rather than TBMN itself.

In 1994, Mochizuki et al. revealed that homozygous or compound heterozygous mutations in *COL4A3* or *COL4A4* cause autosomal recessive AS (ARAS) [22]. Following on from this, Lemmek et al. linked heterozygous *COL4A4* mutation to TBMN patients [8]. Subsequently, several groups confirmed that at least 40% of TBMN was caused by heterozygous *COL4A3* or *COL4A4* mutations [2]. Consequently, some researchers stated that “TBMN/BFH can represent a carrier status of ARAS” [8].

In the late 2000s, researchers from Cyprus showed that 14–35% of TBMN patients with a heterozygous *COL4A3* or *COL4A4* gene mutation developed ESRD later in life, accompanied by pathological findings of FSGS [3, 4, 23]. Therefore, they concluded that renal prognosis of TBMN is not necessarily benign and that the term “BFH” is a misnomer.

### Establishment of the concept of ADAS

AS is a hereditary nephritis caused by the mutation of *COL4A3/A4/A5* genes which encode α3/α4/α5 chains of type IV collagen. Approximately 80% of AS is X-linked AS (XLAS) and typical male XLAS patients present with hearing loss, hematuria and proteinuria in childhood, which progresses to ESRD in the 2nd to 3rd decade of life [1]. ARAS accounts for 15% and presents with similar clinical phenotypes to male XLAS, with equal frequency and severity in males and females [24]. In contrast, ADAS accounts for < 5% and its disease concept has been unclear until recently.

In 1927, Alport AC reported a family of hereditary nephropathy accompanied by deafness, which was named AS after his death in 1961 [25]. Whilst the mode of inheritance was thought to be predominantly X-linked, as males were more severely affected, some researchers argued an autosomal dominant inheritance mode, as there was a transmission from affected father to son [26]. In late 1960s, characteristic changes in the GBM using EM was observed [27]. Subsequently, Flinter et al. described a diagnostic criteria based on clinical symptoms and the appearance of GBM in 1988 [28], aiming to facilitate the identification of classical AS, namely, XLAS.

In 1990, a mutation in *COL4A5* was detected in families with XLAS [29] and subsequently a mutation in *COL4A3* or *COL4A4* was detected in families with ARAS [22]. At that time, typical pattern of lack of α5 chains of type IV collagen [α5 (IV)] by immunohistochemical (IHC) analysis was reported in patients with XLAS and ARAS [30, 31]. Some researchers also reported positive staining for α5 (IV) in patients with autosomal dominant inheritance mode [32].

In 1997, Jefferson et al. provided the first evidence of ADAS, identifying heterozygous mutation in *COL4A3* in a family from Northern Ireland [5]. Out of seven patients, one male patient developed ESRD at 35 years accompanied by deafness. Kidney biopsies from four patients showed irregular thickening of GBM. At that time, as described above, Lemmik. et al. reported that heterozygous mutation in *COL4A3* or *COL4A4* also causes TBMN/BFH and that the same gene mutation either homozygosis or compound heterozygosis can cause ARAS [8]. Therefore, they stated that these gene mutations show various spectra ranging from TBMN/BFH, ARAS, and ADAS [5, 8], leading to the proposal of the concept of “Type IV collagen-related nephropathy”.

After the first report by Jefferson et al., other families with ADAS were reported mostly from Europe [9, 33, 34]. However, since not all patients underwent kidney biopsy and most patients did not have extrarenal manifestations, some experts questioned if all these patients should have been diagnosed with ADAS. In 2016, Kamiyoshi et al. reported genetic, clinical, and pathologic backgrounds of ADAS, using the largest cohort of 72 patients from 16 families [6]. The median age at detection of proteinuria was 17 years and 13% developed ESRD at the median age of 70 years. Hearing loss and ocular abnormalities were reported in 4% of the patients. Light microscopy of kidney biopsies showed non-specific lesions including minor glomerular abnormality, mesangial proliferation, and FSGS. IHC analysis of α5(IV) showed normal expression in all patients. EM analysis showed 44% of the patients with thinning, but without lamellation of the GBM. Therefore, they concluded that it is difficult to make a definitive diagnosis of ADAS based on clinicopathological findings and genetic analysis is essential for an accurate diagnosis. Furthermore, even within one family, clinical severity differed significantly and some individuals developed ESRD, whereas others showed no urinary abnormality. No genotype–phenotype correlations were observed, and no modifier genes were identified among the known podocyte-related genes. These results established the concept of ADAS, and also revealed that both “TBMN developing ESRD” and “ADAS” are caused by *COL4A3* or *COL4A4* heterozygous mutations.

Due to the increased availability of NGS, the number of reports of ADAS is rapidly increasing [9, 10, 34]. For example, Yamamura et al. performed genetic analysis for 390 families with suspicion of AS and showed that XLAS represented 74% of the cases, ARAS 9%, and ADAS 17%, which was notably higher than previously reported [10]. However, some experts still question the diagnosis of ADAS [14].

### Discussion regarding the diagnosis of heterozygous *COL4A3* or *COL4A4* gene variants

**Fig. 1:**
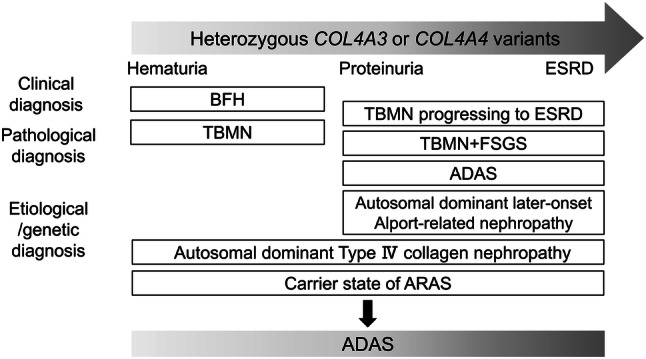
The phenotype of heterozygous *COL4A3* or *COL4A4* gene variants is widely varied and they have been given various diagnostic terms based on clinical, pathological, genetic, and etiological features, resulting in confusion in diagnosis. Some experts recommend that we classify them as ADAS, so that we do not miss the opportunity to start treatment to delay progression into ESRD

The diagnosis of patients with heterozygous mutations in *COL4A3* or *COL4A4* is controversial [7, 13–17, 35], resulting in confusion among nephrologists. This is primarily because the phenotype of heterozygous *COL4A3* or *COL4A4* variants varies from no urinary abnormality, to isolated hematuria, to ESRD. In addition, these patients are given various diagnostic terms based on clinical, pathological, genetic, and etiological features (Fig. 1). Besides the diagnostic terms “TBMN”, “BFH”, and “ADAS” described previously, some patients are classified as “dual diagnosis of FSGS and TBMN” or are even mistaken as “familial FSGS”, when FSGS is pathologically proven [36]. Also, some researchers use the term “Autosomal dominant later-onset Alport-related nephropathy” [35], or “Autosomal dominant collagen IV-related nephropathy” [37]. As the report of patients with heterozygous *COL4A3* or *COL4A4* variants rapidly increases, there are ongoing discussions between the experts regarding the diagnosis of this condition [7, 13–17, 35].

Currently, many researchers believe that the term “TBMN” is not appropriate to describe this condition. This is because “TBMN” is a purely pathological description and implies good clinical outcomes because the term “TBMN” has been used synonymously with “BFH” for a long period. However, renal prognosis for these cases is unpredictable, unless clinicopathological features are observed for a long period. It must also be noted that pathological TBMN can be found in young patients with AS in any inheritance mode [6, 38]. Therefore, some experts insist that diagnosis of TBMN may underestimate the risk of ESRD and deprive these patients of the early treatment.

The diagnostic term ADAS is controversial. Some experts do not accept using the term ADAS [13–15]. This is because most individuals with heterozygous *COL4A3* or *COL4A4* mutations show only hematuria with GBM thinning without progressive renal dysfunction and deafness, nor ocular abnormalities. Therefore, it is considered that these individuals do not fulfill the criteria for AS, and they believe that diagnosis of ADAS will increase anxiety in individuals who actually have low risks of ESRD. Furthermore, the term autosomal dominant implies that half of the affected individuals’ offspring develop AS and ESRD; however, this is not true in heterozygous cases. Actually, even between individuals in a family with the same variants, some family members develop ESRD while some are carriers without any urinary abnormalities. Therefore, they insist that kidney dysfunction might be explained by secondary factors such as modifier genes, coincidental other renal diseases, or other acquired factors including hypertension, diabetes, and obesity [2, 14, 23, 35]. However, to date, how these factors influence patients with ADAS remains largely unknown. Furthermore, they also stated that ADAS should not be used for the carrier status of ARAS because it is not consistent with the practice for other hereditary diseases. For example, in cases with polycystic kidney disease (PKD), ADPKD is caused by the mutations in *PKD1* or *PKD2* genes, whereas ARPKD is caused by mutations in *PKHD1* gene. Thus, ADPKD and ARPKD are independent diseases. On the other hand, ADAS and ARAS are caused by mutations in the same genes and they are not completely independent diseases. In other words, when both parents have heterozygous *COL4A3* or *COL4A4* variants, some of their children can develop ARAS and there can be both patients with ADAS and ARAS in the same families. These relationships between ADAS and ARAS are different from other genetic diseases including PKD. In general, carrier status of “autosomal recessive” diseases are not diagnosed as “autosomal dominant” diseases. Therefore, some experts insist that diagnostic term ADAS will cause confusion in clinical settings. Although they recognize that TBMN is not a satisfactory term, they continue to use this term as it is already commonly accepted worldwide [14, 15].

In contrast, many experts recommend the use of the term ADAS in patients with at least hematuria. Since this condition is caused by heterozygous mutations in *COL4A3* or *COL4A4* and is transmitted in an autosomal dominant fashion, these experts believe that these patients should be diagnosed with ADAS. Although it progresses slower compared to XLAS or ARAS, some patients with ADAS still develop ESRD. Consequently, they insist that diagnosis of ADAS will increase the number of patients who will receive appropriate monitoring and treatment. Of note, some experts have recently proposed a new classification for AS and have stated that individuals with heterozygous mutations in *COL4A3* or *COL4A4* should be classified as ADAS, including patients previously diagnosed as TBMN/BFH [17]. Adding to the confusion among nephrologists, however, is that other experts insist that it is preferable to wait for a full understanding of the factors determining renal prognosis before changing terminology [14, 15].

For most nephrologists, especially in adult section who are not currently familiar with this condition, it is difficult to accept the current complicated situation regarding the diagnosis of this condition. For example, there are some patients who are diagnosed as TBMN at a young age; however, this diagnosis is replaced by ADAS after kidney dysfunction becomes evident later in life. In such cases, it is difficult to determine when patients are TBMN and from when they are ADAS. Therefore, we are very hopeful that there soon will be a consensus with a simple and easy-to-follow classification. From nephrologists’ perspective, we believe that diagnostic term ADAS is significant as most nephrologists still have a strong fixed idea that “TBMN is benign”, misleading our clinical practice. On the other hand, diagnosis of ADAS allows us to follow up these patients carefully. Therefore, we suggest that the common term “ADAS” is used for all cases with heterozygous mutation for *COL4A3* or *COL4A4* with urine abnormality.

### Importance for nephrologists to recognize patients with heterozygous *COL4A3* or *COL4A4* variants

It is important for nephrologists to recognize this disease and current confusion in diagnosis. We recently reported that 69% of cases with adult nephritis accompanied by GBM thinning or thickening who were unable to receive accurate diagnosis clinicopathologically were diagnosed as ADAS by genetic analysis [12]. Furthermore, in a recent paper with a large cohort, *COL4A3* or *COL4A4* gene variants contributed to 16% of 312 cases with CKD [11]. These findings indicate the existence of many undiagnosed patients with ADAS. This is because diagnosis of ADAS is difficult as they lack typical features for AS. Therefore, it is important for nephrologists to recognize this condition and perform genetic analysis for appropriate patients.

Although the indication of genetic analysis is different between countries, NGS before invasive tissue biopsy is already available in several countries. However, genetic testing is still generally not performed when patients and family members present with only hematuria. This is because genetic tests for cases with isolated hematuria can increase anxiety in individuals who actually have low risk of ESRD and also increase medical costs. Therefore, indication of genetic tests should be considered carefully. Currently, genetic analysis is recommended when patients have hematuria with a family history of kidney dysfunction, or when patients have mild to moderate proteinuria or kidney dysfunction, with a family history of hematuria. In sporadic cases, genetic analysis is recommended when kidney biopsy showed GBM thinning and/or lamellation. It is true, however, that it is still common to perform kidney biopsy prior to genetic analysis in patients suspected of having ADAS, due to a lack of recognition of this condition. In fact, there are many patients with AS who are misdiagnosed with FSGS, non-immunoglobulin A (IgA) mesangial proliferative glomerulonephritis, or even familial IgA nephropathy [36, 39], resulting in unnecessary kidney biopsies or immunosuppressive therapies. Therefore, it is important to consider genetic analysis in patients with glomerulonephritis accompanied by GBM thinning and/or lamellation, or even in patients diagnosed with FSGS or IgA nephropathy but are resistant to conventional treatments or with a familial history of hematuria or ESRD. It should also be noted that conditions regarding genetic test for AS are different between countries. For example, it is covered by health insurance in Australia. In the US, sponsored, no-charge genetic testing is performed. On the other hand, it is conducted under laboratory study levels in Japan, Korea, Germany, and many other countries. Therefore, there is still a need to improve genetic testing systems to be widely used as a diagnostic tool to make accurate diagnosis of these cases.

Diagnosis of ADAS is significant because it facilitates careful follow-up and early treatment. Recent large retrospective studies reported that the treatment with angiotensin-converting enzyme inhibitors (ACEIs) delayed the progression into ESRD, even in cases of male XLAS or ARAS (40). Consequently, it is expected that treatment with ACEIs will be even more effective in delaying the progression of ESRD in ADAS patients, who have milder phenotypes compared to male XLAS or ARAS. Therefore, individuals and their family members with heterozygous *COL4A3* or *COL4A4* gene variants, even when they have only isolated hematuria, require annual monitoring of blood pressure, urine protein excretion, and renal function. When proteinuria or hypertension is detected, treatment with renin–angiotensin system inhibitors should commence immediately [13, 17]. It is also true that health checkup system is different between countries. However, even in countries where annual checkup is not easily available, it is recommended to follow these patients at least once per year, if possible. We also have to note that some new medicines have initiated clinical trials, such as bardoxolone methyl (Phase II/III) and RG-012 (effect on microRNA-21 interference, Phase II) [7, 16]. These novel therapies for AS, including ADAS, might change future treatment options.

## Conclusion

Currently, the diagnosis of heterozygous *COL4A3* or *COL4A4* gene variants is complicated. We believe that the diagnosis of ADAS is significant because it facilitates careful follow-up and early treatment and hope that classification of these conditions will be simplified soon. It is important for nephrologists to understand current confusion in diagnosis and the existence of many undiagnosed patients, and to perform genetic analysis for appropriate patients so that adequate treatment can be provided to delay the decline of renal function.
